# Social media use and perceptions of physical health

**DOI:** 10.1016/j.heliyon.2018.e00989

**Published:** 2019-01-08

**Authors:** Bridget Dibb

**Affiliations:** University of Surrey, 28 AD 02, Elizabeth Fry Building, Stag Hill Campus, Guildford Surrey SU2 7XH, UK

**Keywords:** Psychology

## Abstract

Social networking activity is becoming more endemic in society and yet little is known about how the social comparison, occurring when we use these sites, affects perceptions of health. This study sought to determine in what way people who use Facebook (FB) interpret the comparison information they see on FB and whether this was associated with perceptions of physical health. Determining this association is important given the positive association between well-being, quality of life and physical health. Using a cross-sectional design, participants completed an electronic questionnaire measuring FB use, FB social comparison, self-esteem, depression, anxiety, life satisfaction and physical health. The data was analysed using Hierarchical Linear Regression to determine the association of social comparison on perceptions of physical health after controlling for other influencing factors. The results showed that participants were indeed socially comparing via FB. More positive upward comparison was reported, followed closely by positive downward and negative upward, with negative downward comparison perceived least. Analysis showed physical symptoms were associated with gender, anxiety, depression, FB use and positively interpreted upward comparison. Those who agreed more with the positively interpreted social comparison statements and who engaged more with FB also perceived more physical symptoms. These results showed that the perception of symptoms still occurred despite the positive comparison. These results have implications for perceptions of well-being for general users of FB and for vulnerable populations where more social comparison may occur.

## 1 Introduction

Social networking sites have become increasingly popular and provide prime material for social comparison to take place (Haferkamp and Kraemer, 2011; Lee, 2014). Our knowledge of the effects of social comparison via social media is growing but as yet are focused more on perceptions of mental health (Jordan et al., 2011; Kross et al., 2013; Lee, 2014). Social comparison with others can lead to both positive and negative effects over time, depending on the interpretation of the comparison information (Van der Zee et al., 2000). Evidence shows that perceptions of physical functioning can shift as a result of these comparisons (Dibb and Yardley, 2006). Given the strong association of physical functioning and well-being these comparisons may indirectly influence well-being. This study sought to understand the relationship between these comparisons on social media and perceptions of physical health.

Social comparison is a process where we compare ourselves with others in order to evaluate and to self-enhance (Festinger, 1954; Taylor and Lobel, 1989). It is something we all engage in and we do so more when we feel uncertain about our situation (Festinger, 1954; Schachter, 1959). Upward comparison occurs when we compare with someone who we perceive as ‘better-off’ than ourselves, and downward comparison occurs when we compare with someone we feel is ‘worse-off’ than ourselves (Festinger, 1954). Further developments of social comparison theory have shown that the resultant affect experienced after comparing depends more on the interpretation of the comparison rather than on the direction of the comparison (Buunk et al., 1990). A positive interpretation of this comparison has been linked to positive outcomes and a negative interpretation has been linked to negative outcomes (Buunk et al., 1990; Dibb and Yardley, 2006; Van der Zee et al., 2000). For example, comparison with a worse-off target could be interpreted positively (positive downward comparison) where the individual feels ‘lucky’ that they are better-off, or it could be interpreted negatively (negative downward comparison) where the individual may experience anxiety that they too may become like the worse-off target (Buunk et al., 1990). The opposite is true for upward comparison. Here the individual may feel hope and inspiration that they, too, may be like the better-off target (from a positively interpreted comparison; positive upward comparison) or they may feel dejected and depressed if they feel they can never be like the better-off target (after a negatively interpreted comparison; negative upward comparison).

Previous work in social comparison also shows that the valence of the interpretation can have long-term effects, where negatively interpreted comparison can have a long-term negative impact on how we perceive ourselves and our lives (Dibb and Yardley, 2006; Van der Zee et al., 2000). Over identification with the worse-off other can lead to feelings of dejection which can be associated with reduced perceptions of quality of life and lower self-esteem over time (Buunk et al., 1990; Dibb and Yardley, 2006). Self enhancement occurs only when the comparison information is interpreted positively (Buunk et al., 1990; Schachter, 1959).

A pilot study by Gibbons and Gerrard (1989) showed how social comparison can also influence the perceptions of physical health. In this study women who compared themselves, in-person, with a wheelchair bound individual reported fewer physical symptoms than those who compared themselves with an individual who appeared able-bodied. The authors concluded that the comparison with someone with a serious physical disability shifted the perspective of the participants so that their own symptoms were no longer perceived as physical complaints. This study shows the potentially powerful effects that comparing with others can have on our own perceptions of physical health. However, while this study suggests positive effects of downward social comparison, it is not clear what the effects of upward comparison may be. Determining whether comparison with those better-off influences perceptions of our own health is important as perceptions of physical health, well-being and quality of life are positively correlated (Brazier et al., 1992; Garratt et al., 1993; Hernandez et al., 2018; Ropers and Boyer, 1987).

Studies showing the importance of the interpretation of social comparison include those looking at coping and perceptions of quality of life. A positive interpretation was shown to be associated with more active and positive coping in people with cancer (Van der Zee et al., 2000). Another study looking at social comparison and adjustment to living with Meniere's disease showed that positive downward comparison was associated with better perceptions of physical functioning while negative upward comparisons was associated with reduced perceived levels of physical functioning (Dibb and Yardley, 2006).

However, the research discussed above did not include social media as a means of social comparison. Social comparison can occur in many situations, for example, in-person, hearing about others from stories told by well-meaning friends, reading about others in the paper or books, observing others in person, and, of course, the media, including social media (Lee, 2014). However, social comparison using social media is likely to be a little different to social comparison which takes place face-to-face (Vogel et al., 2015). Research has shown that social comparison via social media differs from other types of social comparison as the comparison information seen by others is positively skewed (Appel et al., 2016; Chou and Edge, 2012; Ellison et al., 2006; Gonzales and Hancock, 2011). People tend to post positive attractive photographs and write about happy and successful occurrences and this means that the material is usually upward comparison material and the comparer may view themselves more negatively as a result (Appel et al., 2016). In addition, the target may be of higher relevance for the comparer as the targets are usually friends or acquaintances of the comparer, and a higher relevance usually means that more comparison may occur (Appel et al., 2016).

Research into socially comparing via social media shows that this comparison is linked to depressive symptoms (Appel et al., 2016; Steers et al., 2014; Tandoc et al., 2015), lower perceptions of well-being (Chen et al., 2016; Kross et al., 2013; Verduyn et al., 2015), increased social anxiety (Shaw et al., 2015) and a decrease in self-esteem (Chou and Edge, 2012). Self-esteem was also found to be negatively associated with comparison frequency in students (Lee, 2014). However, we cannot be sure of the long-term effects as these studies were cross-sectional and none of these studies included measures of physical health. With the growing dependency some have for these networking sites and with the amount of time that people spend on these sites increasing, a clearer understanding of the effects of social media use is needed. There are no studies that have looked at the effects of comparison on social media and perceptions of physical health and this is an important point to consider given the strong association that physical health has with quality of life and well-being (Brazier et al., 1992; Garratt et al., 1993; Hernandez et al., 2018; Ropers and Boyer, 1987). This study sought to determine the relationship between the interpretation of social comparison information on social media sites and perceptions of physical symptoms while controlling for psychosocial factors, such as, anxiety, depression, self-esteem, and life satisfaction.

Hypothesis 1 More downward social comparison is associated with the perception of fewer physical symptoms.

Hypothesis 2 More upward comparison is associated with the perception of more physical symptoms.

## 2 Materials and methods

### 2.1 Design

A cross sectional electronic survey design was used to explore the relationship between socially comparing on Facebook (FB) and perceptions of physical health in terms of reported physical symptoms.

### 2.2 Participants

One hundred and sixty five participants completed the questionnaire (a G*Power analysis showed that a sample of 123 would be sufficient to complete the analysis). All participants were over 18 years of age and were current FB users. The mean age was 31.4 years (SD = 13.95; range 18–70) and there were 55 males (33.3%) and 110 females (66.7%). Participants were all members of the general public recruited through FB. One hundred and thirty two participants (80%) considered themselves to be of European decent, 9 (5.5%) of Aisan descent, 16 (9.7%) of African descent, 6 (3.6%) considered themselves to be of mixed race, and 3 (1.8) selected ‘other’ (9 declined to answer).

### 2.3 Procedure

After ethical permission was received from the School of Social Sciences Research Ethics Committee a link to the electronic questionnaire (on SurveyMonkey) was posted on FB inviting participation and inviting the individual to share the post, so engaging in a snowball recruitment technique. FB was used as the focus of this study as it is the most commonly used social media site with over 2 billion users (Tandoc et al., 2015). Participants then followed the instructions and completed the questionnaire which took about 15 minutes. Informed consent was gained at the beginning of the questionnaire.

### 2.4 Measures

Demographic information, such as, age, gender (male = 1; female = 2), and ethnicity were situated at the beginning of the questionnaire.

‘FB use’ was measured using the Facebook Intensity Scale (Ellison et al., 2007) which consists of 8 items, 7 items scored on a 5-point scale (e.g. FB is part of my routine) and 1 item scored on an 8-point scale (number of friends). A composite score was calculated. A high score indicates more engagement with FB.

Social comparison on FB was measured using the Identification/Contrast scale (Van der Zee et al., 2000) modified to include the words ‘on Facebook’, for example, ‘When I see others on Facebook who are experiencing fewer problems than I am, I realise that it is possible to improve’. The scale consists of 12 items which make 4 subscales measuring the four different directions and resultant affect: positive downward comparison, negative downward comparison, negative upward comparison and positive upward comparison. Responses were recorded on a 5-point Likert scale; strongly disagree to strongly agree. A high score indicates more agreement with the interpretation of the comparison statement.

Self-esteem was measured with Rosenberg's (Rosenberg, 1965) Self-esteem scale which consists of 10 items all scored on a 4-point scale ranging from strongly agree to strongly disagree (for example, I feel that I have a number of good qualities.). A high score indicates higher self-esteem.

Depression and Anxiety were measured with the Hospital Anxiety & Depression Scale (HAD) (Zigmond and Snaith, 1983) which consists of two subscales (Depression (for example, I still enjoy the things I used to enjoy) and Anxiety (for example, I feel tense or ‘wound up’)) with 7 items each, all rated on a 4-point scale measuring frequency of symptoms. A high score indicates more depression or anxiety.

Perceived physical health was measured with the Cohen-Hoberman Inventory of Physical Symptoms (CHIPS) (Cohen and Huberman, 1983) which lists 39 common physical symptoms over the past 2 weeks and asks the individual to rate how much they were bothered by the symptom on a 5-point scale (not at all to extreme bother). Examples of the 39 symptoms include sleep problems, weight change, fatigue and muscle tension. A high score indicates more perceived physical symptoms (worse physical health).

Life satisfaction was measured with the Satisfaction with Life scale (SWLS) (Diener et al., 1985) which consists of 5 items rated on a 7-point scale (strongly disagree to strongly agree), scored so that a high score indicates more satisfaction with life. An example of one of the items is ‘In most ways my life is close to my ideal.’

### 2.5 Analysis

Descriptive statistics were used to assess the sample characteristics and hierarchical linear regression analysis was used to determine the relationship between the independent and dependent variables. A square root transformation was performed on the outcome measure, Physical Symptoms, as it was skewed (Field, 2013). In order to determine whether social comparison on FB was associated with the perception of physical health after controlling for other variables, variables were entered in three steps. The demographic variables (age and gender) were entered at step 1, the psychosocial variables (life satisfaction, self-esteem, anxiety and depression) at step 2, and the social comparison variables (positive and negative upward and downward comparison, and FB use) at step 3.

## 3 Results

The sample characteristics are presented in Table 1 below. The means show the average self-esteem score to be high, which indicates high self-esteem (Rosenberg, 1965) and anxiety and depression to be low in this sample, indicating reduce feelings of depression and anxiety (Zigmond and Snaith, 1983). With regard to comparison, more people agreed with the positively interpreted comparison statements than with the negatively interpreted comparison statements. In addition, more agreement was evident with positively interpreted upward comparison followed by positively interpreted downward comparison and negatively interpreted upward comparison, with negatively interpreted downward comparison experienced the least.

**Table 1:** Means, standard deviations and alpha values of the variables.

Variable	Mean (SD)	Range	α
Self esteem	30.63 (5.58)	15–40	.90
Anxiety	7.81 (4.01)	0–19	.83
Depression	3.91 (2.80)	0–12	.68
Reported symptoms	23.9 (19.29)	0–99	.93
Life satisfaction	25.31 (5.75)	7–35	.86
Positive upward comparison	9.07 (2.43)	3–15	.82
Positive downward comparison	8.90 (2.34)	3–15	.67
Negative downward comparison	6.30 (2.35)	3–15	.86
Negative upward comparison	8.30 (3.14)	3–15	.85
FB use (item mean)	2.83 (3.70)	1–5	.84

Just under half the sample had over 400 friends on FB and about half the sample were using FB for under an hour (Table 2).

**Table 2:** Frequencies (percentages) of the number of friends on FB and the time spent on FB.

FB Use	N (Percentage)
Number of friends on FB	
>10	3 (1.8)
11–100	15 (9)
101–200	27 (16.4)
201–300	28 (17)
301–400	18 (10.9)
>400	74 (44.8)
Time spent on FB per day	
Less than 10 minutes	28 (17)
10–30 minutes	29 (17.6)
31–60 minutes	27 (16.4)
1–2 hours	40 (24.2)
2–3 hours	20 (12.1)
More than 3 hours	21 (12.7)

The bivariate correlations between the variables are presented in Table 3 below. Younger participants reported more physical symptoms (worse physical health). Anxiety, depression and negatively interpreted comparison (downward and upward) were all associated with more physical symptoms (worse physical health). However, higher scores in life satisfaction and self-esteem were associated with fewer physical symptoms (better physical health). FB use was associated with age, showing that younger people were more engaged with FB. Positive downward comparison was also associated with more FB use. Negatively interpreted comparison was associated with more anxiety, depression and more physical symptoms (worse physical health).

**Table 3:** Pearson correlation matrix of study variables.

	1	2	3	4	5	6	7	8	9	10	11
1. Age	1										
2. Lifesatisfaction	.155^∗^	1									
3. Self-esteem	.266^∗∗^	.594^∗∗^	1								
4. Anxiety	−.318^∗∗^	−.441^∗∗^	−.643^∗∗^	1							
5. Depression	−.075	−.548^∗∗^	−.601^∗∗^	.554^∗∗^	1						
6. FB use	−.358^∗∗^	.082	−.033	.107	−.068	1					
7. Positive downward comparison	−.141	.131	.128	−.050	−.029	.220^∗∗^	1				
8. Negative upward comparison	−.377^∗∗^	−.445^∗∗^	−.570^∗∗^	.601^∗∗^	.407^∗∗^	.136	.216^∗∗^	1			
9. Positive upward comparison	−.065	.043	.156^∗^	−.061	.064	.022	.573^∗∗^	.023	1		
10. Negative downward comparison	−.091	−.109	−.385^∗∗^	.400^∗∗^	.384^∗∗^	.121	.171^∗^	.529^∗∗^	.145	1	
11. Physical symptoms	−.234^∗∗^	−.437^∗∗^	−.444^∗∗^	.578^∗∗^	.482^∗∗^	.130	−.008	.318^∗∗^	.144	.186^∗^	1

Note: *p < .05; **p < .01.

The hierarchical linear regression results are presented in Table 4. The final model accounted for 47.3% of the variance (F (11,150) = 12.242, p < .000). At Step 1 age and gender are significantly associated with physical symptoms with younger participants and female participants reporting more physical symptoms. At Step 2, after the introduction of the psychosocial variables, age ceases to be important but gender maintains a positive association. In addition, life satisfaction is significantly associated with physical symptoms, where those with low life satisfaction report more physical symptoms. Anxiety and depression also prove to be important with significant positive associations with physical symptoms. This suggests that those who are more anxious and have more depressive symptoms also perceive more physical symptoms. Finally, for Step 3, gender continues to maintain the positive relationship with physical symptoms showing that women perceive more physical symptoms after entering all the other variables into the equation. Anxiety and depression also maintain a positive association with physical symptoms indicating that high scores in anxiety and depression are associated with worse physical health. After controlling for demographic and psychosocial variables only upward comparison demonstrates a significant relationship and, in particular, only positively interpreted upward comparison reached significance. This suggests that those who agreed more with items about positive feelings after comparison with someone better-off also perceived more physical symptoms. Neither positive and negative downward comparison nor upward negative comparison were significantly associated with physical health. Finally, FB use also had a positive significant relationship with physical symptoms showing that those who feel more that FB is part of their lives also perceived more physical symptoms.

**Table 4:** Hierarchical Linear Regression results for variables associated with reported symptoms.

	R^2^ (total R^2^)	Unstandardized		Standardised		Sig.
		B	SE	B	T	
**Step 1**	.078 (.473)					.002
Age		−.018	.007	−.209	−2.722	.007
Gender		.406	.192	.162	2.115	.036
**Step 2**	.341 (.473					.000
Age		−.005	.006	−.056	−.855	.394
Gender		.319	.156	.127	2.039	.043
Self esteem		.009	.019	.045	.486	.628
Life Satisfaction		−.034	.016	−.164	−2.047	.042
Anxiety		.110	.026	.370	4.243	.000
Depression		.097	.036	.229	2.684	.008
**Step 3**	.054 (.473					.011
Age		−.001	.006	−.007	−.104	.918
Gender		.335	.153	.134	2.194	.030
Self esteem		−.011	.020	−.051	−.527	.599
Anxiety		.124	.027	.418	4.628	.000
Depression		.087	.037	.206	2.379	.019
Life Satisfaction		−.034	.017	−.164	−1.939	.054
FB use		.030	.014	.139	2.086	.039
Positive upward		.110	.037	.226	2.961	.004
Negative upward		−.034	.036	−.091	−.944	.346
Positive downward		−.046	.041	−.091	−1.130	.260
Negative downward		−.040	.039	−.080	−1.024	.307

## 4 Discussion

This study aimed to progress our understanding of the influence of social media on perceptions of physical health. In particular, it aimed to determine whether perceptions of physical health are associated with socially comparing with others, particularly with others on FB/social media. The hypotheses predicted that more downward social comparison would be associated with the perception of fewer physical symptoms (Hypothesis 1) and more upward comparison would be associated with the perception of more physical symptoms (Hypothesis 2).

These results confirm that social network users do engage in a range of comparisons including both upward and downward comparison. However, for Hypothesis 1, although the direction of the relationship was as expected, this relationship was not significant and so was not supported. Hypothesis 2 was partially supported; those who engaged more with positive upward comparison also perceived more physical symptoms (reduced health) and this association was significant. However, as negative upward comparison was not significantly associated with physical symptoms this hypothesis was only partially supported. These results contribute to the existing knowledge of the negative effects of comparison, for example, low self-evaluation after upward comparison (Haferkamp and Kraemer, 2011) and targets perceived as better off and happier (Chou and Edge, 2012). With regard to FB use, those who felt FB was an important part of their lives also perceived more physical symptoms. The increased FB use may be associated with more opportunities to view others who appear to have fewer symptoms.

These results indicate that more positive upward comparison was associated with the perception of symptoms and this contributes to our understanding of comparing on social media. That participants agreed with upward comparison statements more supports studies showing that people tend to post positive messages and photos and so are more likely to be viewed as upward targets (Dorethy et al., 2014; Fardouly et al., 2017).

These results also show that positive upward social comparison, while using social media, can have both positive and negative associations. The positive upward comparison relationship in this study shows that the participants were feeling hopeful and inspired but at the same time were aware of worse physical health. This supports social comparison theorists who have shown that upward comparison is associated with hope and optimism where the comparer feels inspired to become more like the target (Taylor and Lobel, 1989). However, these results also suggest the possible negative effects of positive upward comparison while using social networking sites, as more physical symptoms were perceived.

Social Comparison theorists may propose that those in poorer health are engaging in positive upward comparison and may be gaining knowledge and experiencing hope from these comparisons (Taylor and Lobel, 1989). The positive interpretation coupled with increased physical symptoms may also be testament to different dimensions of comparison. Social comparison can occur on different dimensions, for example, coping style or financial success etc. (Gibbons, 1999). The scale measuring social comparison in this study asked questions about ‘doing well’ or ‘not doing so well’ in relation to others with more or fewer ‘difficulties’ and it was not specific about which dimension the difficulties were related to. As comparison can occur on different dimensions it is possible that the participants did not compare within a physical health dimension. Instead they may have compared on a dimension unrelated to physical health and which did not interfere with the positive interpretation of the comparison measured in this study, so resulting in a significant association between positive upward comparison and physical symptoms (reduced physical health).

It is also possible that those who had more physical symptoms tended to engage in more positive upward comparison to be more like the better-off target. This may be a coping strategy and would account for why those engaging in upward comparison would also be more aware of their symptoms. A longitudinal study would best show the true relationships and the direction of causality; does social comparison lead to perceptions of ill health or are those who experience worse health more likely to engage in positive upward comparison? The answer to this question is important in order to determine the long-term negative effects of comparing via social media.

Future research could also investigate the possible mechanisms of this association. One possible mechanism may be rumination where individuals mull over and dwell on past events (Feinstein et al., 2015). Research has shown that there is an association between upward comparison and rumination (Jordan et al., 2011). Rumination has also been found to occur when individuals use social media, however, this has not been looked at in the context of perceptions of physical health (Tandoc et al., 2015).

The limitations of this study include the cross-sectional design which limits the conclusions we can draw about causality. These results are also based on those who used FB and so may not be relevant for those not using FB and who engage with other social networking sites. Another limitation is that the type of target and the dimension of the comparison was not measured. Future studies would also benefit from including measures of social network activity as those who actively post or comment on others' posts may interpret the information differently to those who simply observe others' posts.

In conclusion, social comparison on FB and strong feelings that FB was an important part of life were associated with worse physical health. The knowledge of how using social media sites influences us is important given that although there can be positive effects, there is also the danger of negative effects. Given the positive association between physical health, well-being and quality of life, there is a potential for long-term negative effects to result from social networking. Further investigation is needed to determine these long-term effects.

## Declarations

### Author contribution statement

Bridget Dibb: Conceived and designed the experiments; Performed the experiments; Analyzed and interpreted the data; Contributed reagents, materials, analysis tools or data; Wrote the paper.

### Funding statement

This research did not receive any specific grant from funding agencies in the public, commercial, or not-for-profit sectors.

### Competing interest statement

The authors declare no conflict of interest.

### Additional information

No additional information is available for this paper.
